# Hierarchically ordered mesoporous Co_3_O_4_ materials for high performance Li-ion batteries

**DOI:** 10.1038/srep19564

**Published:** 2016-01-19

**Authors:** Shijiao Sun, Xiangyu Zhao, Meng Yang, Linlin Wu, Zhaoyin Wen, Xiaodong Shen

**Affiliations:** 1 College of Materials Science and Engineering, Nanjing Tech University, Nanjing, 210009, China; 2 CAS key laboratory of Materials for Energy Conversion, Shanghai Institute of Ceramics, Chinese Academy of Sciences, Shanghai, 200050, China

## Abstract

Highly ordered mesoporous Co_3_O_4_ materials have been prepared via a nanocasting route with three-dimensional KIT-6 and two-dimensional SBA-15 ordered mesoporous silicas as templates and Co(NO_3_)_2_ · 6H_2_O as precursor. Through changing the hydrothermal treating temperature of the silica template, ordered mesoporous Co_3_O_4_ materials with hierarchical structures have been developed. The larger pores around 10 nm provide an efficient transport for Li ions, while the smaller pores between 3–5 nm offer large electrochemically active areas. Electrochemical impedance analysis proves that the hierarchical structure contributes to a lower charge transfer resistance in the mesoporous Co_3_O_4_ electrode than the mono-sized structure. High reversible capacities around 1141 mAh g^−1^ of the hierarchically mesoporous Co_3_O_4_ materials are obtained, implying their potential applications for high performance Li-ion batteries.

Co_3_O_4_ was developed as anode in lithium ion batteries in 2000 by Poizot *et al.*^1^. It can theoretically uptake more than 8 lithium per formula unit with a capacity as high as 890 mAh g^−1^. However, the major drawbacks of quick capacity fading upon extended cycling and/or poor rate capability hinder the practical use of bulk Co_3_O_4_. A key causation could be related to the large volume change during lithium insertion/deinsertion, which leads to pulverization of the material and loss of electrical contact, eventually causes failure of the electrode^2^,^3^. To solve this problem, Co_3_O_4_ with different nanostructures, such as one-dimensional (1D) nanotubes^2^, nanorods and nanobelts^4^, two-dimensional (2D) nanosheets^5^ and nanomeshs^6^, three-dimensional (3D) nanocubes^7^, nanoflowers^8^ and nanocages^9^, have been investigated as the negative electrode materials for lithium ion batteries. Ordered mesoporous structure (composed of micrometer-sized particles containing nanometer diameter pores separated by walls of similar size) is one of the most appealing nanostructures for Li-ion batteries. In general, ordered mesoporous structure can be synthesized by either the soft template or hard template method through a process called nanocasting. Up to now, various ordered mesoporous materials such as lithiated metal oxides (LiCoO_2_^10^ and LiMn_2_O_4_ 11), carbon^12^ and transition metal oxides (Cr_2_O_3_ 13, SnO_2_ 14, CuCo_2_O_4_ 15) have been employed as the electrode materials for Li-ion batteries. There are several advantages of ordered mesoporous electrodes for lithium ion batteries. Micrometer-sized particles can result in good interparticle contact, hence high packing density and volumetric energy density^16^. Meanwhile, the mesoporous structure can enhance the access of Li^+^ in the electrolyte to the electrode surface and provide better accommodation for the volume changes^3^. The nanosized pore wall can shorten the diffusion distance for lithium ion^14^. Furthermore, the well-ordered mesoporous materials can facilitate ionic motion as compared with conventional mesoporous materials in which the pores are randomly connected^11^. Preliminary results by Wang *et al.*^17^ have shown that ordered mesoporous Co_3_O_4_ is an efficient anode material for lithium storage.

Herein, ordered mesoporous Co_3_O_4_ with tunable textural parameters were synthesized by using KIT-6 and SBA-15 silicas as templates. Through changing the hydrothermal treating temperature of the KIT-6 template, ordered mesoporous Co_3_O_4_ materials with hierarchical structures have been developed. The lithium storage behaviors of these hierarchically mesoporous Co_3_O_4_ materials were evaluated for the first time. Moreover, the electrochemical impedance analysis was conducted to account for the different electrochemical behavior on ordered mesoporous Co_3_O_4_ with different textural parameters. Besides, we compared the lithium storage capabilities between ordered mesoporous Co_3_O_4_ with 2D hexagonal symmetry and ordered mesoporous Co_3_O_4_ with 3D cubic symmetry.

## Results and Discussion

### Material characterization

**Figure 1:**
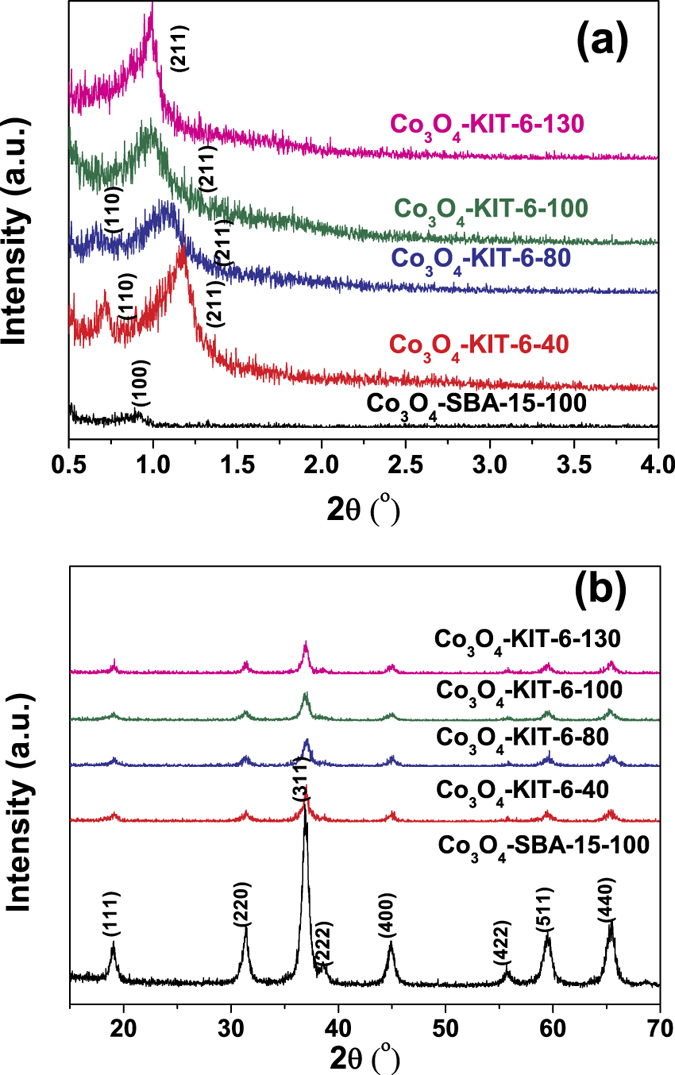
(**a**) Low-angle and (**b**) wide-angle X-ray diffraction (XRD) patterns of ordered mesoporous Co_3_O_4_.

Low-angle XRD patterns indicate that all the samples are ordered mesoporous (Fig. 1a). For the products nanocast from KIT-6, both Co_3_O_4_-KIT-6-100 and Co_3_O_4_-KIT-6-130 exhibit one well-defined diffraction peak indexed as (211). They possess the same mesoscopic symmetry as their parent silicas with space group *Ia*3*d*, indicating that the mesostructures of their parent silicas were duplicated. The other two products (Co_3_O_4_-KIT-6-40 and Co_3_O_4_-KIT-6-80) exhibit two well-defined diffraction peaks indexed as (110) and (211) with space group *I*4_1_32^18^. Their mesoscopic symmetries are lower than those of their parent silicas. For Co_3_O_4_-SBA-15-100, a relatively small peak was displayed, which can be indexed as (100) with space group of *P*6*mm*. Hence, Co_3_O_4_-SBA-15-100 possesses the same 2D hexagonal symmetry as its template. Wide-angle powder X-ray diffraction results (Fig. 1b) show that all phases are coincident, demonstrating that the face centered cubic spinel structure dominates the wall of the mesoporous solid.

**Figure 2 f2:**
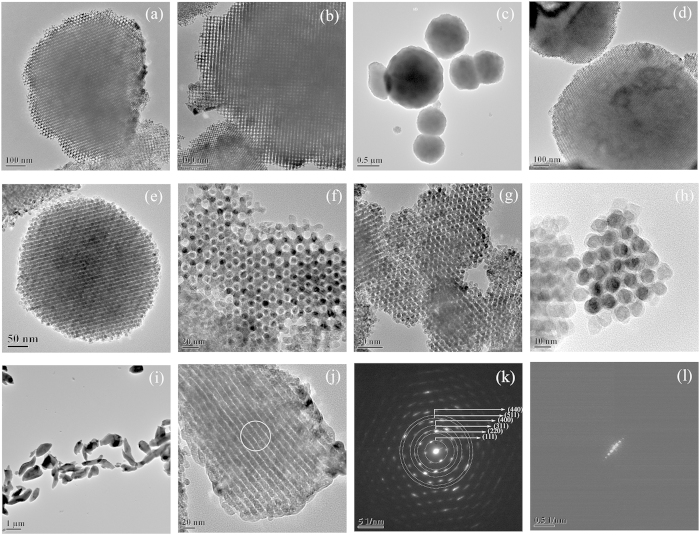
(**a**–**h**) TEM images with different magnifications of different ordered mesoporous Co_3_O_4_ materials nanocast from KIT-6: (**a**,**b**) Co_3_O_4_-KIT-6-40; (**c**,**d**) Co_3_O_4_-KIT-6-80; (**e**,**f**) Co_3_O_4_-KIT-6-100; (**g**,**h**) Co_3_O_4_-KIT-6-130; (**i**) A general profile and (**j**) a high magnification TEM image of ordered mesoporous Co_3_O_4_-SBA-15-100 and the corresponding (**k**) SAED pattern and (**l**) FFT pattern.

All the samples were analyzed by Transmission electron microscopy (TEM), which confirmed the highly ordered mesoporous structure (Fig. 2). Conventional cobalt oxide particles were not observed for all the samples from TEM observation. This indicates that almost all nitrates have moved into the mesopores of silicas during the calcination. Figure 2a–h shows the TEM images with different magnifications of the mesoporous Co_3_O_4_ materials nanocast from KIT-6. The square image contrast pattern of Co_3_O_4_-KIT-6-40 (Fig. 2b), where the mesoporous channels are seen as bright contrast, indicating the image is viewed down the [100] zone axis of KIT-6 related cubic unit cell. Most particles of mesoporous Co_3_O_4_-KIT-6-80 (Fig. 2c) were spherical in shape with a particle size ranging from 0.64 to 1.30 μm, indicating the crystal growth in a 3D mesoporous system. The TEM images in Fig. 2f[Fig f2] g are viewed along the [111] and [311] zone axis of KIT-6 related cubic unit cell, respectively^19^. Figure 2i–l shows the TEM images and the corresponding selected area electron diffraction (SAED) of the mesoporous Co_3_O_4_-SBA-15-100 material, which exhibits a worm-like overall morphology (Fig. 2i). A magnified view of a mesoporous Co_3_O_4_ bundle (Fig. 2j) shows the presence of mono-dimensional aligned channels between two aligned nanorods. According to the literature^20^, adjacent Co_3_O_4_ nanorods are connected by Co_3_O_4_ spacers formed inside SBA-15 micropores. The SAED pattern of the area marked with a circle in Fig. 2j is shown in Fig. 2k; the ring-like diffraction pattern indicates the nanocrystalline walls of the mesoporous Co_3_O_4_-SBA-15-100. Fast Fourier transform (FFT) pattern in Fig. 2l is simply an inverse form of the entire nanowire bundle, in which the spots reflect the highly ordered arrangement of parallel nanowires. Energy-dispersive X-ray (EDX) spectra of all the mesoporous Co_3_O_4_ materials confirm no trace of Si, which means that the silica templates have been completely removed.

**Figure 3:**
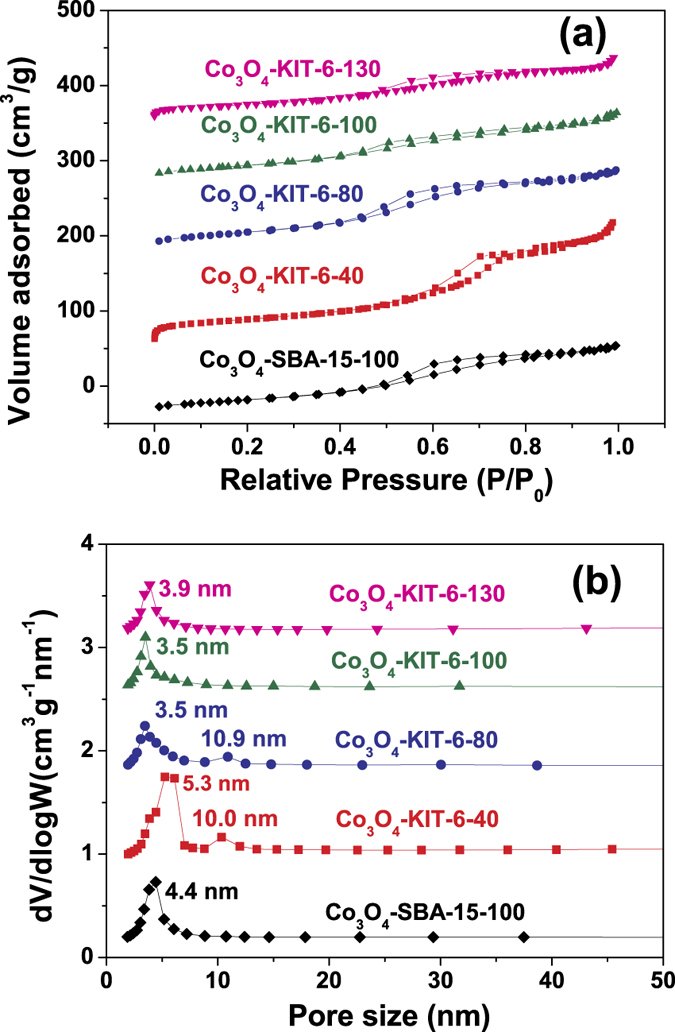
(**a**) N_2_ adsorption-desorption isotherms and (**b**) BJH pore size distributions for different ordered mesoporous Co_3_O_4_ materials.

Figure 3 shows the N_2_ adsorption-desorption isotherms and (Barrett-Joyner-Halenda) pore size distribution plots of mesoporous Co_3_O_4_. Typical IV adsorption-desorption isotherms with H1-type hysteresis are observed for all the samples. This is ascribed to the formation of mesoporosity. Moreover, the capillary condensation range is broad for all the sorption isotherms starting at about *P*/*P*_0_ = 0.4 and extending almost to *P*/*P*_0_ = 0.9. This indicates that all the samples have a high fraction of textural porosity^21^. The BJH pore size distributions show that mesoporous Co_3_O_4_-KIT-6-40 and Co_3_O_4_-KIT-6-80 have a bimodal pore-size distribution, which are centered at 5.3/10 nm and 3.5/10.9 nm, respectively. The smaller pore size of 5.3 or 3.5 nm reflects the minimum wall thickness of KIT-6, while larger pore size of 10 or 10.9 nm is corresponding to the wall junctions in KIT-6^22^. Whereas, the other two mesoporous Co_3_O_4_ materials have a unimodal pore-size distribution, with the pore size of 3.5 nm for Co_3_O_4_-KIT-6-100 and 3.9 nm for Co_3_O_4_-KIT-6-130. It is well known that KIT-6 possesses two sets of mesoporous systems, which are connected by micropores. The amount of micropores depends on the temperature of hydrothermal treatment. When KIT-6 was treated at lower temperature such as 40 or 80 °C, a part of the two mesoporous systems were not connected. Accordingly, hierarchically porous structure was obtained; While KIT-6 was treated at higher temperature such as 100 or 130 °C, the two mesoporous systems were well interconnected. Accordingly, porous structure with mono-sized pores was obtained. Textural properties of these samples were summarized in Table 1. The BET surface areas and pore volumes of the hierarchically mesoporous Co_3_O_4_ materials (Co_3_O_4_-KIT-6-40 and Co_3_O_4_-KIT-6-80) are larger than those of the other two mesoporous Co_3_O_4_ materials with mono-sized pores (Co_3_O_4_-KIT-6-100 and Co_3_O_4_-KIT-6-130).

### Electrochemical properties

**Figure 4:**
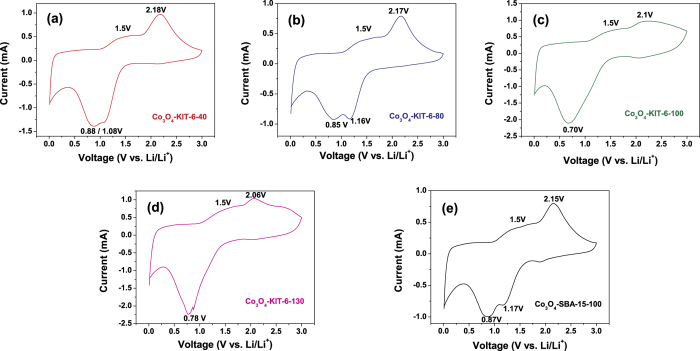
CV curves of the ordered mesoporous Co_3_O_4_ electrodes at a scan rate of 0.5 mV s^−1^.

Figure 4 shows the cyclic voltammetry (CV) curves of the ordered mesoporous Co_3_O_4_ electrodes at a scan rate of 0.5 mV s^−1^ in the second cycle. When the electrodes were scanned cathodically, for Co_3_O_4_-KIT-6-40, Co_3_O_4_-KIT-6-80 and Co_3_O_4_-SBA-15-100, two reduction peaks (Li insertion) located between 0.85 V and 1.17 V appeared, corresponding to the reduction processes from Co_3_O_4_ to CoO (or Li_x_Co_3_O_4_) and CoO (or Li_x_Co_3_O_4_) to Co, respectively. The reduction of Co_3_O_4_ was accompanied by the formation of Li_2_O. For Co_3_O_4_-KIT-6-100 and Co_3_O_4_-KIT-6-130, only one reduction peak emerged; this is because the two reduction peaks which should appear merge together. During the following anodic polarization, one broad hump at around 1.5 V and one sharp peak at around 2.1 V were observed for all the mesoporous Co_3_O_4_, which is corresponding to the reverse process where Co is reoxidized to Co_3_O_4_ and Li_2_O is decomposed^23^. Furthermore, besides the redox peaks, a rectangular shape area related to the reflection by supercapacitor^24^,^25^ is observed at the lower potential in each CV pattern. This indicates that besides the lithium storage according to the conversion reaction of between Co_3_O_4_ and lithium, the electrochemical process by the capacitive contribution is also included.

**Figure 5:**
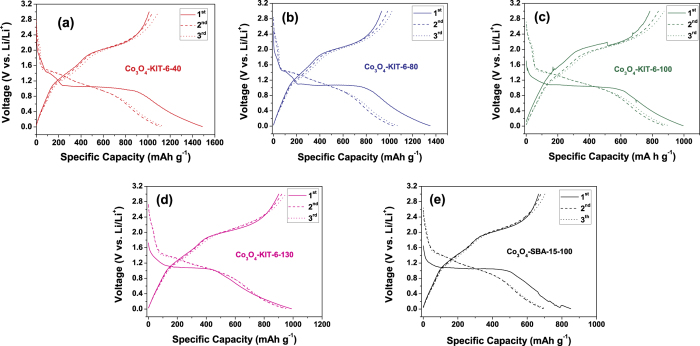
The first three charge-discharge curves of the ordered mesoporous Co_3_O_4_ electrodes at a current density of 50 mA g^−1^ between 0.01 and 3.0 V.

Figure 5 shows the first three charge (delithiation) and discharge (lithiation) curves of ordered mesoporous Co_3_O_4_ electrodes at a current density of 50 mA g^−1^ between 0.01 and 3.0 V. On one hand, in the first cycle, for all of the mesoporous Co_3_O_4_, one clear discharge voltage plateau at around 1.0 V and the corresponding ambiguous charge voltage plateaus at around 2.1 V are observed. Besides, an additional small discharge plateau at around 1.40 V is observed. This may be related to the formation of an intermediate phase between Co_3_O_4_ and metallic cobalt. The intercalated Li_x_Co_3_O_4_ intermediate is always formed upon the early stage of reduction, but its stability is highly dependent on the applied current density. When the current density is low, the Li_x_Co_3_O_4_ intermediate spontaneously decomposes into the CoO intermediate, results in the presence of a high voltage plateau at the initial discharge. Moreover, the mesoporous Co_3_O_4_-KIT-6-40 or Co_3_O_4_-KIT-6-80 material shows a higher surface area and thus the corresponding current density per unit surface area was decreased. Hence, the small plateau around 1.40 V was detected^23^. On the other hand, for all of the mesoporous Co_3_O_4_ electrodes, the discharge voltage plateaus became unconspicuous in the subsequent cycles. This is the typical characteristic of Co_3_O_4_ electrodes^6^,^26^,^27^.

**Figure 6:**
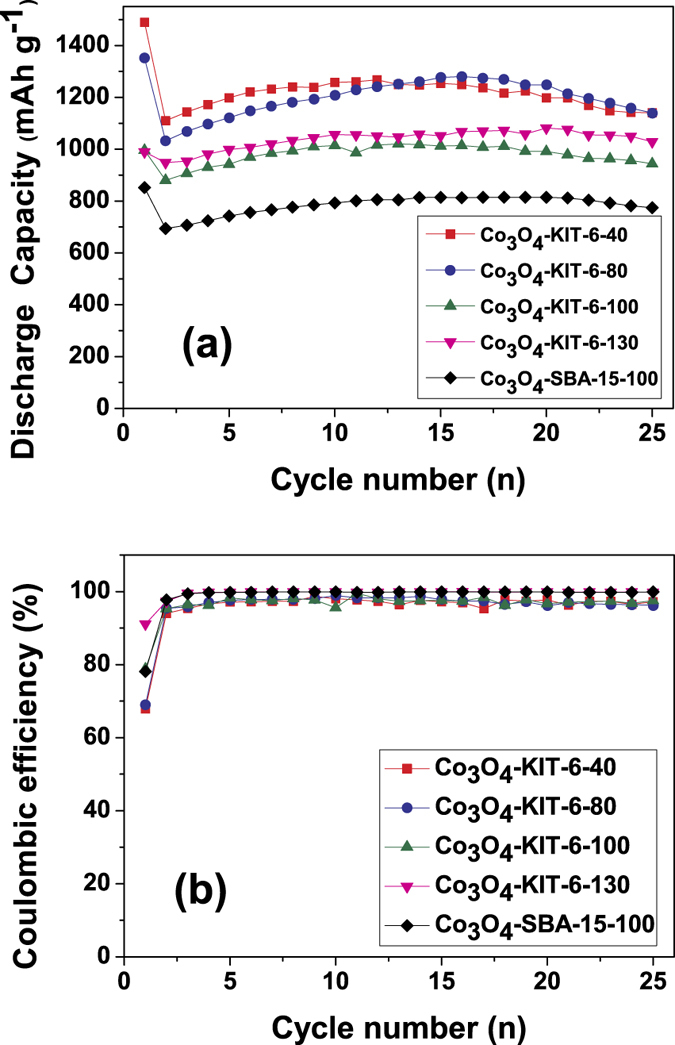
Cycling performance of ordered mesoporous Co_3_O_4_ with different textural parameters at a current density of 50 mA g^−1^: (**a**) discharge capacities versus cycle number; (**b**) coloumbic efficiencies versus cycle number.

Figure 6a shows the variation of discharge capacities versus cycle number for the ordered mesoporous Co_3_O_4_ electrodes cycled between 0.01–3.0 V at the current density of 50 mA g^−1^. For all of the Co_3_O_4_ electrodes, they demonstrate superior cycling stability. The discharge capacity gradually increases upon initial cycles, especially for the Co_3_O_4_-KIT-40 and Co_3_O_4_-KIT-6-80 with hierarchically mesoporous structure. Similar phenomenon has been also observed on Co_3_O_4_ nanomaterials^26^,^27^,^28^,^29^,^30^. We could not explicitly explain this phenomenon. The higher surface areas of our mesoporous materials might be responsible for this behavior. The electrolyte needs some time to access the inner surface within the mesopores to establish stable electric double layer. Hence, the gradual formation of the electric double layer in the mesopores could be the reason. Furthermore, the following two points can be drawn from Fig. 6a. Firstly, the hierarchically mesoporous Co_3_O_4_-KIT-6-40 and Co_3_O_4_-KIT-80 deliver higher discharge capacities than the mesoporous Co_3_O_4_-KIT-6-100 and Co_3_O_4_-KIT-6-130 with mono-sized pores throughout the 25 cycles. We ascribe the better Li storage properties to their relatively larger BET surface areas, pore volumes and the presence of additional large pores around 10 nm, which are favorable for Li ion transport^31^. Secondly, Co_3_O_4_-KIT-6-100 exhibits superior performance than Co_3_O_4_-SBA-15-100, although the pore size and pore volume of Co_3_O_4_-KIT-6-100 is lower than those of Co_3_O_4_-SBA-15-100 (Table 1). This implies that the 3D cubic *Ia*3*d* mesoporous structure makes the infiltration of the liquid electrolyte more facile than the 2D hexagonal *P*6*mm* mesoporous structure. Besides, coulombic efficiencies are evaluated and shown in Fig. 6b. For all of the ordered mesoporous Co_3_O_4_, except for the relatively low initial coulombic efficiencies (67.9–91.1%) typical for conversion reaction^32^, the coulombic efficiencies in the subsequent cycles almost maintain above 95%, indicating their excellent electrochemical reversibility. The first discharge capacities together with those after 25 cycles for these mesoporous Co_3_O_4_ electrodes are given in Table 2. The as-prepared Co_3_O_4_ materials deliver high initial discharge capacities between 852–1489 mAh g^−1^. After 25 cycles, the discharge capacities still maintain at a high level of 774–1141 mAh g^−1^. Note that these mesoporous Co_3_O_4_ electrodes exhibit capacities higher than the theoretical capacity of Co_3_O_4_ (890 mAh g^−1^). This phenomenon is very common for Co_3_O_4_ nanostructure^4^. These large excess capacities could be ascribed to lithium storage in the interconnected mesopores via an electric double layer capacitive mechanism, showing sloping discharge profiles at low potential in Fig. 5. Meanwhile, a rough performance comparison with other forms of Co_3_O_4_ nanostructures reported before was summarized in Table 2. The as-prepared mesoporous Co_3_O_4_ electrodes show comparable and/or even superior Li storage performance, which could be ascribed to their hierarchically ordered mesoporous structures. It has been demonstrated that the large surface area of the ordered mesoporous electrodes can decrease the current density per unit surface area, and the thin wall of ordered mesoporous electrodes can reduce the length of the Li^+^ diffusion path. Moreover, compared with conventional mesoporous materials in which the pores are randomly connected, the well-ordered mesoporous materials can facilitate ionic motion more easily^11^. Most importantly, the hierarchical structure provides not only efficient transport channels for Li ions but also large electrochemically interface. Hence, the current hierarchically mesoporous Co_3_O_4_ could be the choice of anode material for Li-ion batteries.

**Figure 7:**
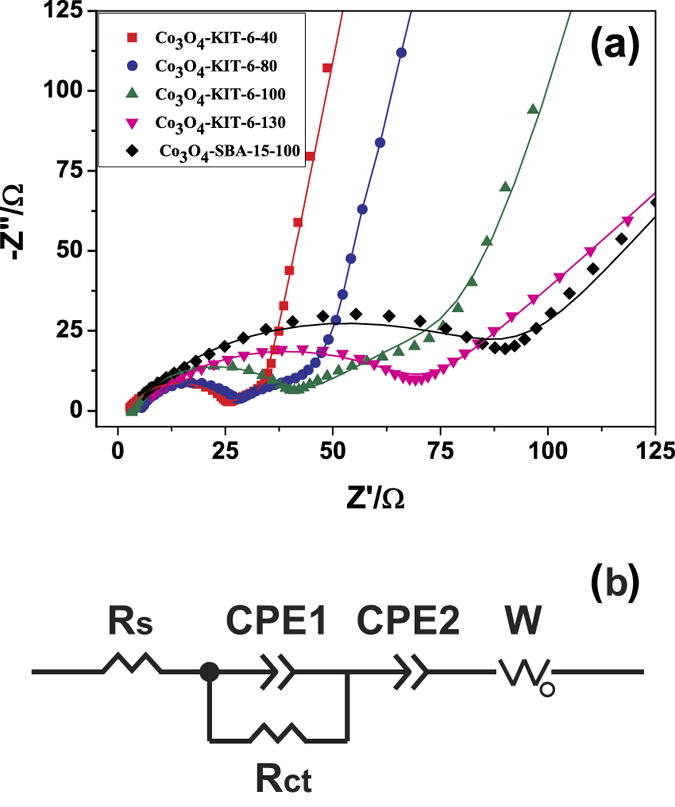
(**a**) Nyquist plots of the fresh ordered mesoporous Co_3_O_4_ electrodes measured at the open potential over the frequency range from 10^5^ Hz to 0.01 Hz with the amplitude of 5 mV and (**b**) the corresponding equivalent circuit.

In order to account for the different electrochemical behaviors of the as-prepared ordered mesoporous Co_3_O_4_ electrodes, electrochemical impedance tests were conducted. The Nyquist plots of the fresh Co_3_O_4_ electrodes measured at the open potential are shown in Fig. 7a. For the Co_3_O_4_-KIT-6-40, Co_3_O_4_-KIT-6-80 and Co_3_O_4_-KIT-6-100 electrodes, a depressed semicircle in the high-frequency region and an arc in the medium-frequency region are observed. However, for the Co_3_O_4_-KIT-6-130 and Co_3_O_4_-SBA-15-100 electrodes, the semicircle in the high-frequency region and the arc in the medium-frequency region overlap together. Besides, for all the Co_3_O_4_ electrodes, a slopping line was found in the low-frequency region. In order to interpret the measured results, an equivalent circuit model (Fig. 7b) was used to fit the Nyquist plots. The diameter and intercept of the semicircle at the *Z*’ axis in the high-frequency region represent charge transfer resistance (*R*ct) and electrolyte resistance (*R*s), respectively, among which *R*ct accounts for a large proportion of the overall kinetic impedance of the cell. Constant phase elements (CPE1 and CPE2) are related to the double layer capacitive effect. The sloping line in the low-frequency region represents the Warburg impedance (W), which reflects the solid-state diffusion of Li^+^ within the bulk anode^33^. The fitted charge transfer resistances (*R*ct) were summarized in Table 3. The hierarchically mesoporous Co_3_O_4_ electrodes possess smaller *R*ct (20 Ω for Co_3_O_4_-KIT-6-40 and 21 Ω for Co_3_O_4_-KIT-6-80) than the other mesoporous Co_3_O_4_ electrodes with mono-sized pores (33 Ω for Co_3_O_4_-KIT-6-100, 65 Ω for Co_3_O_4_-KIT-6-130 and 91 Ω for Co_3_O_4_-SBA-15-100). Hence, ordered mesoporous Co_3_O_4_ with hierarchical structure is more favorable for Li ion transport, which is consistent with the discussion above. Besides, ordered mesoporous Co_3_O_4_-KIT-6-100 exhibits smaller *R*ct than Co_3_O_4_-SBA-15-100, which further confirms the 3D cubic *Ia*3*d* mesoporous structure makes the transport of Li ion more facile than the 2D hexagonal *P*6*mm* mesoporous structure.

## Conclusion

Textural parameters of ordered mesoporous Co_3_O_4_ can be regulated by varying the hydrothermal treating temperatures of the KIT-6 template. When KIT-6 hydrothermally treated at a lower temperature of 40 °C or 80 °C was employed as the template, well-ordered mesoporous Co_3_O_4_ materials with hierarchical structures were obtained, showing the signature of a bimodal pore-size distribution and larger BET specific surface area and pore volume. These hierarchical mesoporous Co_3_O_4_ materials exhibit superior Li storage performance than the mesoporous Co_3_O_4_ with mono-sized pores due to their smaller charge transfer impedances. Besides, 3D cubic mesoporous Co_3_O_4_ is more beneficial for Li ion storage than 2D hexagonal mesoporous Co_3_O_4_. Reversible discharge specific capacities around 1141 mAh g^−1^ were obtained over the hierarchically porous Co_3_O_4_ materials at a current density of 50 mA g^−1^, which are comparable with or even higher than those reported in the literature. Hence, the as-prepared well-ordered mesoporous Co_3_O_4_ with hierarchical structure could be the promising anode materials for high performance Li-ion batteries.

## Methods

### Synthesis of KIT-6 and SBA-15 silica

3D cubic *Ia*3*d* KIT-6 mesoporous silica materials were prepared according to the procedure described by Ryoo and co-workers^34^. In a typical synthesis, 6 g of P123 was dissolved in 217 mL of distilled water with 10 mL of conc. HCl (37 wt%). 7.41 mL of n-butanol was added to the mixture under stirring at 35 °C. Then, this mixture was stirred for 1 h at 35 °C before 13.87 mL of TEOS was added. After stirring at 35 °C for another 24 h, the mixture was subsequently transferred into stainless-steel autoclaves, followed by the hydrothermally treated at 100 °C for 24 h. The resulting mixture was filtered without washing and dried at 80 °C. The organic template was removed by calcination at 550 °C for 6 h in air at a heating rate of 3 °C min^−1^. The product was nominated as KIT-6-100 (“100” denotes the hydrothermal treating temperature of KIT-6). In another set of experiments, the hydrothermal treating temperature was varied from 40 °C to 130 °C.

2D hexagonal *P*6*mm* SBA-15 mesoporous silica material was synthesized according to the literature^35^ with the hydrothermal treating temperature of 100 °C. The product was nominated as SBA-15-100.

### Synthesis of mesoporous Co_3_O_4_

Mesoporous Co_3_O_4_ was prepared via the “two solvent” method^36^ using calcined 3D cubic KIT-6 and 2D hexagonal SBA-15 as templates. Typically, 1.0 g of as-prepared silica was suspended in 40 mL of dry n-hexane. After stirring for 3 h at room temperature, 1.0 mL of 1.5 g mL^−1^ Co(NO_3_)_2_ · 6H_2_O aqueous solution as the second solution was added dropwise under vigorous stirring. The mixture was stirred overnight. Then, a pink powder specimen was obtained by filtration and dried at room temperature. The solid was then calcined in a muffle furnace with a heating rate of 1 °C min^−1^ from room temperature to 300 °C and maintained at this temperature for 5 h. The silica template was removed by etching twice with heated 2 M NaOH aqueous solution for 12 h each time. The black Co_3_O_4_ material was collected by filtering, washing with water and ethanol, and then dried at 80 °C. When KIT-6 and SBA-15 were used as templates, the products were nominated as Co_3_O_4_-KIT-6-T (“T” denotes the hydrothermal treating temperature of KIT-6) and Co_3_O_4_-SBA-15-100, respectively.

### Materials characterization

Powder X-ray diffraction (XRD) patterns were recorded on a Rigaku D/max 2200 X-ray diffractometer using Ni-filtered Cu Kα radiation (λ = 0.15418 nm) operating at 40 kV and 40 mA. Transmission electron microscopy (TEM) images were measured on a JEOL JEM-2010 transmission electron microscope equipped with an Oxford energy-dispersive X-ray (EDX) spectrometer attachment operating at 200 kV. Nitrogen adsorption-desorption isotherms were measured on a Micromeritics ASAP 2020M analyzer at liquid nitrogen temperature (77 K). Prior to determination of the isotherm, the samples were degassed at 423 K in vacuum for 5 h. The Brunauer-Emmett-Teller (BET) specific surface area was calculated using the adsorption data in the relative pressure (*P*/*P*_0_) range from 0.05 to 0.3, and the total pore-volume was determined from the amount adsorbed at *P*/*P*_0_ = 0.98. The pore-size distribution curve was calculated based on the desorption branch of the isotherm using the Barrett-Joyner-Halenda (BJH) method. The pore diameter was defined as the position of the maximum in the pore-size distribution.

### Electrochemical Test

Electrochemical performance of the powders was evaluated with two-electrode CR2032-type coin cells with a lithium foil counter electrode and an electrolyte consisting of a 1 M LiPF_6_ solution in EC/DMC (1:1 by volume). Microporous polypropylene membrane (celgard 2400) was used as the separator. The working electrode was constructed from a paste consisting of 75% active powder, 15% conductive acetylene black and 10% PVDF binder in NMP solvent. The paste was cast onto Cu foil and finally dried at 100 °C under vacuum for 12 h before electrochemical evaluation. The loading weight of the active material on the electrode is about 2 mg. The cell assembly was operated in an argon-filled glove box (VAC AM-2) with oxygen and water contents less than 1 ppm. Cyclic voltammetry (CV) measurement of the electrode was performed between 3.0 and 0.01 V at a scan rate of 0.5 mV s^−1^ using an electrochemical workstation (CHI 604C). The galvanostatic charge and discharge test was carried out using a LAND CT2001A battery test system in the voltage window of 0.01–3.0 V at a current density of 50 mA g^−1^. AC impedance of the cell was measured by a Frequency Response Analyzer (FRA) technique on an Autolab Electrochemical Workstation over the frequency range from 10^5^ Hz to 0.01 Hz with the amplitude of 5 mV. All the electrochemical measurements were conducted at room temperature.

## Additional Information

**How to cite this article**: Sun, S. *et al.* Hierarchically ordered mesoporous Co_3_O_4_ materials for high performance Li-ion batteries. *Sci. Rep.* **6**, 19564; doi: 10.1038/srep19564 (2016).

## Figures and Tables

**Table 1:** Textural parameters of ordered mesoporous Co_3_O_4_.

Sample	BET surface area (m^2^ g^−1^)	Pore size (nm)	Pore volume (cm^3^ g^−1^)
Co_3_O_4_-KIT-6-40	105	5.3/10.0	0.23
Co_3_O_4_-KIT-6-80	87	3.5/10.9	0.15
Co_3_O_4_-KIT-6-100	84	3.5	0.13
Co_3_O_4_-KIT-6-130	69	3.9	0.12
Co_3_O_4_-SBA-15-100	79	4.4	0.14

**Table 2:** Performance comparison of different forms of Co_3_O_4_ nanostructures.

Co_3_O_4_ nanostructures	Current density (mA g^−1^)	1^st^ discharge capacity (mAh g^−1^)	Capacity upon cycling (mAh g^−1^)	References
Co_3_O_4_-KIT-6-40	50	1489	1141 after 25 cycles	This work
Co_3_O_4_-KIT-6-80	50	1352	1140 after 25 cycles	This work
Co_3_O_4_-KIT-6-100	50	995	943 after 25 cycles	This work
Co_3_O_4_-KIT-6-130	50	989	1029 after 25 cycles	This work
Co_3_O_4_-SBA-15-100	50	852	774 after 25 cycles	This work
Mesoporous Co_3_O_4_ nanoflakes	89	1192	883 after 25 cycles	37
Hollow-structured Co_3_O_4_ nanoparticles	50	1107	880 after 25 cycles	38
Co_3_O_4_ mesoporous microdisks	100	1032	765 after 30 cycles	39
Co_3_O_4_ nanocages	178	1116	864 after 50 cycles	9
Co_3_O_4_ nanobowl and nanotube arrays	35	1468, 1293	843, 895 after 10 cycles	40
Co_3_O_4_ nanoparticles with opened-book morphology	100	1408	950 after 25 cycles	41
Co_3_O_4_ nanoflowers	50	1849	980 after 30 cycles	8
Hairy ball-like Co_3_O_4_ nanostructures	100	1768	860 after 50 cycles	42
Wire-like Co_3_O_4_ nanostructures	50	1043	275 after 20 cycles	43
Co_3_O_4_ nanobelt array	177	1086	750 after 25 cycles	26
Co_3_O_4_ nanorods and nanobelts	44.5	1739, 1550	1124, 1260 after 50 cycles	4
Porous Co_3_O_4_ nanorods	50	1518	1132 after 30 cycles	44
Porous Co_3_O_4_ nanorods	50	1171	850 after 10 cycles	45
Co_3_O_4_ nanotubes, nanoparticles and nanorods	50	850, 830 and 815	500, 480 and 450 after 100 cycles	46
Porous Co_3_O_4_ nanotube	50	1918	1131 after 20 cycles	47
Needlelike Co_3_O_4_ nanotube	50	2300	918 after 25 cycles	48

**Table 3:** Fitted charge transfer resistance in the equivalent circuit.

Sample	*R*ct/Ω
Co_3_O_4_-KIT-6-40	20
Co_3_O_4_-KIT-6-80	21
Co_3_O_4_-KIT-6-100	33
Co_3_O_4_-KIT-6-130	65
Co_3_O_4_-SBA-15-100	91
